# Supplementary material for: Modeling glioblastoma heterogeneity as a dynamic network of cell states
Source: Mol Syst Biol. 2021 Sep 16;17(9):e10105. doi: 10.15252/msb.202010105 (PMC8444284; doi:10.15252/msb.202010105)
Supplement: Supplementary file 6 — Source Data for Figure 5 [file MSB-17-e10105-s004.zip › Figure5A_sourcedata/GSEA_3017/nosigdb_stateF.GseaPreranked.1622795015644/LARSSON_STATE_6.html]

Details for gene set LARSSON\_STATE\_6[GSEA]

|  || Dataset | state33017 |
| Phenotype | NoPhenotypeAvailable |
| Upregulated in class | na\_pos |
| GeneSet | LARSSON\_STATE\_6 |
| Enrichment Score (ES) | 0.5937473 |
| Normalized Enrichment Score (NES) | 3.2662663 |
| Nominal p-value | 0.0 |
| FDR q-value | 0.0 |
| FWER p-Value | 0.0 |
Table: GSEA Results Summary

  

Fig 1: Enrichment plot: LARSSON\_STATE\_6      
 Profile of the Running ES Score & Positions of GeneSet Members on the Rank Ordered List

  

| PROBE | GENE SYMBOL | GENE\_TITLE | RANK IN GENE LIST | RANK METRIC SCORE | RUNNING ES | CORE ENRICHMENT || 1 | NEAT1 |  |  | 0 | 2.864 | 0.0656 | Yes |
| 2 | MT3 |  |  | 1 | 2.229 | 0.1167 | Yes |
| 3 | CLU |  |  | 5 | 1.852 | 0.1578 | Yes |
| 4 | SPARCL1 |  |  | 6 | 1.804 | 0.1991 | Yes |
| 5 | ZMAT3 |  |  | 10 | 1.568 | 0.2337 | Yes |
| 6 | HOPX |  |  | 14 | 1.345 | 0.2631 | Yes |
| 7 | LGALS3 |  |  | 16 | 1.261 | 0.2916 | Yes |
| 8 | FTH1 |  |  | 19 | 1.203 | 0.3182 | Yes |
| 9 | S100B |  |  | 20 | 1.167 | 0.3450 | Yes |
| 10 | PNRC1 |  |  | 23 | 1.070 | 0.3686 | Yes |
| 11 | FTL |  |  | 32 | 1.036 | 0.3887 | Yes |
| 12 | TNFRSF21 |  |  | 36 | 1.016 | 0.4106 | Yes |
| 13 | DKK3 |  |  | 37 | 0.994 | 0.4333 | Yes |
| 14 | MXD4 |  |  | 39 | 0.987 | 0.4555 | Yes |
| 15 | SAT1 |  |  | 45 | 0.957 | 0.4751 | Yes |
| 16 | GFAP |  |  | 47 | 0.953 | 0.4965 | Yes |
| 17 | SESN3 |  |  | 56 | 0.908 | 0.5137 | Yes |
| 18 | TSC22D4 |  |  | 64 | 0.855 | 0.5301 | Yes |
| 19 | CRYAB |  |  | 85 | 0.783 | 0.5389 | Yes |
| 20 | PHPT1 |  |  | 109 | 0.738 | 0.5453 | Yes |
| 21 | BRI3 |  |  | 121 | 0.716 | 0.5567 | Yes |
| 22 | FEZ1 |  |  | 133 | 0.691 | 0.5675 | Yes |
| 23 | PBX1 |  |  | 179 | 0.613 | 0.5610 | Yes |
| 24 | GABARAPL2 |  |  | 232 | 0.568 | 0.5502 | Yes |
| 25 | CEBPD |  |  | 234 | 0.566 | 0.5628 | Yes |
| 26 | NLRP1 |  |  | 257 | 0.547 | 0.5652 | Yes |
| 27 | MAGED1 |  |  | 258 | 0.545 | 0.5777 | Yes |
| 28 | OLFM2 |  |  | 343 | 0.490 | 0.5506 | Yes |
| 29 | SCP2 |  |  | 353 | 0.485 | 0.5576 | Yes |
| 30 | ID2 |  |  | 366 | 0.483 | 0.5632 | Yes |
| 31 | EEF1A1 |  |  | 417 | 0.464 | 0.5510 | Yes |
| 32 | MAP1LC3A |  |  | 430 | 0.460 | 0.5561 | Yes |
| 33 | FHL1 |  |  | 467 | 0.445 | 0.5498 | Yes |
| 34 | GABBR2 |  |  | 494 | 0.437 | 0.5480 | Yes |
| 35 | HEY1 |  |  | 496 | 0.436 | 0.5575 | Yes |
| 36 | BEX1 |  |  | 518 | 0.428 | 0.5578 | Yes |
| 37 | CITED1 |  |  | 561 | 0.411 | 0.5480 | Yes |
| 38 | PMP2 |  |  | 562 | 0.411 | 0.5574 | Yes |
| 39 | BNIP3L |  |  | 565 | 0.409 | 0.5659 | Yes |
| 40 | RAB31 |  |  | 580 | 0.404 | 0.5687 | Yes |
| 41 | IRS2 |  |  | 595 | 0.400 | 0.5715 | Yes |
| 42 | PLEKHB1 |  |  | 597 | 0.399 | 0.5802 | Yes |
| 43 | RND2 |  |  | 608 | 0.396 | 0.5847 | Yes |
| 44 | ATP6V1G1 |  |  | 609 | 0.395 | 0.5937 | Yes |
| 45 | ATP6V1F |  |  | 685 | 0.374 | 0.5681 | No |
| 46 | SH3BGRL |  |  | 695 | 0.373 | 0.5725 | No |
| 47 | FABP7 |  |  | 701 | 0.371 | 0.5787 | No |
| 48 | PFN2 |  |  | 724 | 0.365 | 0.5771 | No |
| 49 | TTC3 |  |  | 779 | 0.352 | 0.5605 | No |
| 50 | TPT1 |  |  | 786 | 0.350 | 0.5658 | No |
| 51 | ID3 |  |  | 789 | 0.349 | 0.5728 | No |
| 52 | FOXO3 |  |  | 801 | 0.346 | 0.5758 | No |
| 53 | METRN |  |  | 903 | 0.325 | 0.5371 | No |
| 54 | BEX3 |  |  | 978 | 0.313 | 0.5105 | No |
| 55 | FAM20C |  |  | 1029 | 0.304 | 0.4947 | No |
| 56 | C1orf122 |  |  | 1081 | 0.296 | 0.4782 | No |
| 57 | SNX3 |  |  | 1150 | 0.285 | 0.4537 | No |
| 58 | C4orf48 |  |  | 1209 | 0.278 | 0.4336 | No |
| 59 | CTNNA1 |  |  | 1228 | 0.276 | 0.4317 | No |
| 60 | RTL8C |  |  | 1324 | 0.265 | 0.3944 | No |
| 61 | HNRNPA2B1 |  |  | 1503 | -0.261 | 0.3191 | No |
| 62 | HNRNPDL |  |  | 1940 | -0.448 | 0.1304 | No |
| 63 | FABP5 |  |  | 2033 | -0.523 | 0.1004 | No |
Table: GSEA details [plain text format]

  

Fig 2: LARSSON\_STATE\_6: Random ES distribution      
 Gene set null distribution of ES for **LARSSON\_STATE\_6**
